# Hepatitis E Virus Persistence and/or Replication in the Peripheral Blood Mononuclear Cells of Acute HEV-Infected Patients

**DOI:** 10.3389/fmicb.2021.696680

**Published:** 2021-07-16

**Authors:** Ibrahim M. Sayed, Zeinab A. Abd Elhameed, Doaa M. Abd El-Kareem, Mohamed A. Y. Abdel-Malek, Mohamed E. Ali, Maggie A. Ibrahim, Ayat Abdel-Rahman Sayed, Khaled Abo bakr Khalaf, Lobna Abdel-Wahid, Mohamed A. El-Mokhtar

**Affiliations:** ^1^Department of Medical Microbiology and Immunology, Faculty of Medicine, Assiut University, Assiut, Egypt; ^2^Department of Pathology, School of Medicine, University of California, San Diego, La Jolla, CA, United States; ^3^Department of Clinical Pathology, Faculty of Medicine Assiut University, Assiut, Egypt; ^4^Department of Microbiology and Immunology, Faculty of Pharmacy, Al-Azhar University, Assiut, Egypt; ^5^Department of Medical Biochemistry, Faculty of Medicine, Assiut University, Assiut, Egypt; ^6^Department of Tropical Medicine and Gastroenterology, Faculty of Medicine, Assiut University, Assiut, Egypt; ^7^Gastroenterology and Hepatology Unit, Department of Internal Medicine, Faculty of Medicine, Assiut University, Assiut, Egypt; ^8^Microbiology and Immunology Department, Faculty of Pharmacy, Sphinx University, Assiut, Egypt

**Keywords:** HEV, PBMCs, acute hepatitis, cytokines, gene expression, persistence and replication

## Abstract

**Background:**

Hepatitis E virus (HEV) causes about 14 million infections with 300,000 deaths and 5,200 stillbirths worldwide annually. Extrahepatic manifestations are reported with HEV infections, such as renal, neurological, and hematological disorders. Recently, we reported that stool-derived HEV-1 replicates efficiently in human monocytes and macrophages *in vitro*. However, another study reports the presence of viral RNA but no evidence of replication in the PBMCs of acute hepatitis E (AHE) patients. Therefore, the replication of HEV in PBMCs during AHE infection is not completely understood.

**Methods:**

PBMCs were isolated from AHE patients (*n* = 17) enrolled in Assiut University Hospitals, Egypt. The viral load, positive (+) and negative (−) HEV RNA strands and viral protein were assessed. The gene expression profile of PBMCs from AHE patients was assessed. In addition, the level of cytokines was measured in the plasma of the patients.

**Results:**

HEV RNA was detected in the PBMCs of AHE patients. The median HEV load in the PBMCs was 1.34 × 10^3^ IU/ml. A negative HEV RNA strand and HEV open reading frame 2 protein were recorded in 4/17 (23.5%) of the PBMCs. Upregulation of inflammatory transcripts and increased plasma cytokines were recorded in the AHE patients compared with healthy individuals with significantly elevated transcripts and plasma cytokines in the AHE with detectable (+) and (−) RNA strands compared with the AHE with the detectable (+) RNA strand only. There was no significant difference in terms of age, sex, and liver function tests between AHE patients with detectable (+) and (−) RNA strands in the PBMCs and AHE patients with the (+) RNA strand only.

**Conclusion:**

Our study shows evidence for *in vivo* HEV persistence and replication in the PBMCs of AHE patients. The replication of HEV in the PBMCs was associated with an enhanced immune response, which could affect the pathogenesis of HEV.

## Graphical Abstract

Flow of the study design: Blood collected from acute HEV patients were fractiontated into plasma and PBMCs. Plasma was used for cytokine assay. RNA was extracted from the PBMCs and used for determinaton of viral load, detection of (+) and (−) HEV RNA strands, and gene expression assay.

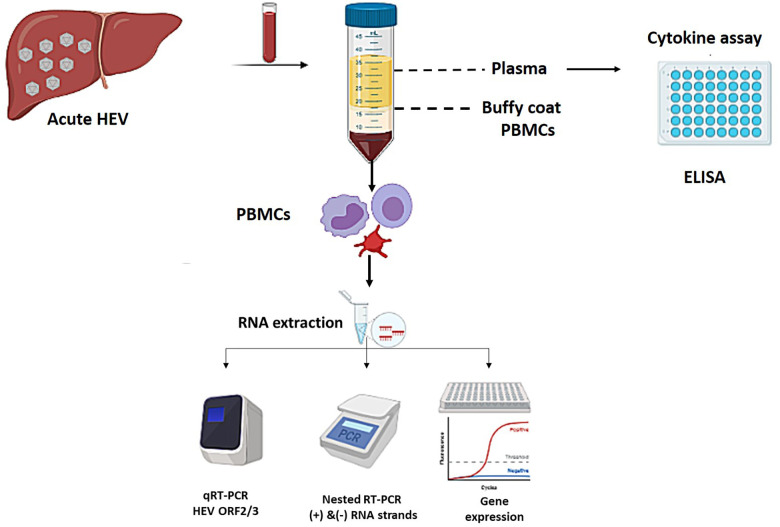

## Introduction

Hepatitis E virus (HEV) is a small icosahedral positive-sense, single-stranded RNA virus. The HEV genome includes three open reading frames (ORF1–3) with a 7-methylguanosine-cap at 5′ end and a poly A-tail at 3′ end (Tam et al., 1991; Sayed et al., 2015a). ORF1 is located at the 5′ end and encodes a non-structural polyprotein with a methyltransferase, Y domain, cysteine protease, hypervariable domains, X domain, RNA helicase, and RNA-dependent RNA polymerase activity (Koonin et al., 1992; Sayed et al., 2015a). ORF2 is located at the 3′ end and encodes the structural capsid protein, which is involved in the viral entry and modulation of the host immune response (Kalia et al., 2009; Hingane et al., 2020). ORF3 encodes a small phosphoprotein that is a functional ion channel required for the release of infectious HEV particles (Ding et al., 2017).

HEV is the most common cause of acute viral hepatitis worldwide; it is estimated that about 15–110 million individuals are experiencing ongoing or recent infection (Li et al., 2020). HEV belongs to the *Hepeviridae* family, and it includes eight genotypes (HEV-1 to HEV-8); five of them cause infection to humans (Smith et al., 2020). HEV-1 and HEV-2 are common in developing countries, and they infect humans through the fecal–oral route (Rein et al., 2012; Hakim et al., 2017). HEV-1 can transmit from HEV-infected mothers to newborns, and it is associated with fatal complications (Sharma et al., 2017). HEV-3 and HEV-4 are common in developed countries (Sayed et al., 2015b). HEV-3, HEV-4, and HEV-7 are zoonotic isolates, and HEV infection can be transmitted through the ingestion of contaminated, undercooked animal products (Colson et al., 2010; Abravanel et al., 2017; Anheyer-Behmenburg et al., 2017; Pavio et al., 2017; El-Mokhtar et al., 2020a; Sayed et al., 2020a). Moreover, the transmission of HEV infection via transfusion of contaminated whole blood or blood products is documented (Hewitt et al., 2014).

HEV causes acute, chronic, and extrahepatic manifestations. Acute hepatitis E (AHE) infection is a self-limiting disease, but progression to fulminant hepatic failure is reported (Péron et al., 2007; Sayed et al., 2016, 2021; El-Mokhtar et al., 2021a). Immunocompromised patients are at high risk for the development of HEV chronicity (Kamar et al., 2008; Kenfak-Foguena et al., 2011). Ribavirin and interferon are used off-label in the treatment of HEV infections (Haagsma et al., 2010; Kamar et al., 2014; Péron et al., 2016). Extrahepatic disorders are reported with HEV infection, such as neurological, renal, hematological, acute pancreatitis, and complications during pregnancy (Pischke et al., 2017; El-Mokhtar and Sayed, 2021). Hematological disorders associated with HEV infection could be linked directly or indirectly with the replication of HEV in the blood cells and/or components. Plasma, granulocytes, platelets, and red blood cells are sources of transfusion-transmitted (TT) HEV infection (Hewitt et al., 2014; Huzly et al., 2014). Data available on the replication of HEV in PBMCs is limited. Recently, our groups reported that human monocytes, macrophages, and bone marrow–derived macrophages are susceptible to infection by stool-derived HEV inoculums *in vitro* (Sayed et al., 2020c). On the other hand, Ippagunta et al. (2011) report the presence of HEV RNA but no evidence of replication in the PBMCs of AHE patients. Till now, the available data on the replication of HEV in PBMCs during AHE infection is limited and not widely studied.

In this study, we assess if HEV replicates in the PBMCs of AHE patients. Also, we evaluate whether virus replication in PBMCs is associated with differences in the immune response to HEV infection, which could impact the severity of disease in those patients.

## Materials and Methods

### Patients

This study includes AHE patients (*n* = 17) admitted to Assiut University Hospital, Assiut Fever Hospital, and AL-Rajhi Liver University Hospital as described (El-Mokhtar et al., 2021a,b; Sayed et al., 2021). The diagnosis of AHE infection is based on clinical and laboratory diagnosis and according to the guidelines of EASL (European Association for the Study of the Liver, 2018). The clinical symptoms include one or more of the following symptoms: jaundice, abdominal pain, fever, dark urine, and pale stool. The laboratory diagnosis of AHE was based on the assessment of liver function tests, such as alanine transaminase (ALT), aspartate transaminase (AST), and bilirubin as well as HEV markers, such as HEV RNA, anti-HEV IgM, anti-HEV IgG, and HEV Ag, and as described before (El-Mokhtar et al., 2021b; Sayed et al., 2021). Blood samples were collected from each subject who provided written consent, and the protocol for assessment of HEV in the blood and/or components was approved by the Institutional Review Board (IRB nos 17200190 and 17300400) at the Faculty of Medicine, Assiut University, Egypt, following the provisions of the Declaration of Helsinki.

### Isolation of Plasma and PBMCs From the Blood Samples

Blood samples were centrifuged at 800 × *g* for 10 min, and the plasma was collected. To isolate the PBMCs, an equal volume of blood was mixed with PBS containing 2% fetal bovine serum and layered over Ficoll-Paque Plus and centrifuged at 800 × *g* for 30 min. Then, we collected the buffy coat layer and spun at 100 × *g* for 15–20 min at room temperature to pellet the PBMCs. The PBMC pellets were washed several times with PBS and centrifuged again to precipitate the PBMC pellets.

### Quantification of HEV RNA

HEV RNA was extracted from plasma and PBMCs using the RNeasy Mini Kit (Qiagen, Germany). HEV RNA was detected and quantified by RT-qPCR using primers targeting the HEV ORF2/3 region as described before (Sayed et al., 2020b). Details of the RT-qPCR are described in Supplementary Material and Methods.

Sequencing of HEV was performed using primers targeting HEV ORF2. The sequences of AHE samples were deposited in the Genbank and assigned the following numbers: MW888849-MW888852, MW924820-MW924826.

### Detection of the Positive (+) and Negative (−) RNA Strands in the PBMCs

HEV RNA was assessed in the PBMCs of AHE patients by strand-specific nested RT-PCR using primers targeting HEV ORF1 for (+), (−) RNA strands. We used the methodology first described by Chatterjee et al. (2012) and modified by us (Sayed et al., 2017b; Montpellier et al., 2018). We adapted the protocol for the specific detection of (+) and (−) sense HEV RNA strands in the PBMCs enrolled in the study. Briefly, extracted RNA was converted into cDNA using the Superscript III enzyme (Life Technologies) with primers specific for either positive- or negative-sense RNA as described before (Chatterjee et al., 2012; Sayed et al., 2017b). Then, we treated the cDNA with exonuclease I (Life Technologies) and purified it using the Wizard SV gel and PCR Clean-Up (Promega). Primers used for amplification are listed in Supplementary Table 1. Details about the PCR reaction condition are mentioned in Supplementary Material and Methods. Plasma samples from AHE patients served as positive controls for the (+) RNA strand and as a negative controls for the (−) RNA strand. Total intracellular RNA extracted from HEV-infected primary monocyte cells were used as positive controls for both (+) and (−) RNA strands (Sayed et al., 2020c). We used these cells because anti-ds RNA was recorded inside the cells when challenged with HEV inoculums (Sayed et al., 2020c). The PBMC RNA load was lower than the plasma control sample, thereby ensuring that the signal of the (−) RNA strand was not caused by a specific amplification of excess (+) RNA strand.

### Detection of HEV ORF2 Protein in the PBMCs of AHE Patients

Detection of HEV ORF2 Ag in the PBMCs of AHE patients was done as described previously (El-Mokhtar et al.,2020b,c; Sayed et al., 2020c). Briefly, the PBMCs were fixed and permeabilized using eBioscience^TM^ Fixation/Permeabilization Concentrate (Thermo Fischer Scientific, United States), and the cells were stained with anti-HEV ORF2 protein (1E6 clone, Millipore) that targets amino acids 434–457. The secondary antibody used was goat antimouse IgG conjugated with Alexa488 (Invitrogen) according to the manufacturer’s instructions. PBMCs from healthy controls were processed by the same methodology and served as a negative control.

### Measurement of the Cytokine Transcript Level in the Infected PBMCs

Total cellular RNA was extracted from the PBMCs as mentioned previously, and RNA was converted into cDNA using MultiScribe reverse transcriptase according to the manufacturer’s instructions (Invitrogen, California) as described before (El-Mokhtar et al., 2020b). RT-qPCR was carried out using SYBR green master mix (Applied Biosystems, Foster City, California, United States) on 7500 Fast Real-Time PCR (Applied Biosystems) for target genes and normalized to the housekeeping gene (β-actin) using the 2^–ΔΔ*Ct*^ method. The sequences of primers used in this study are listed in Supplementary Table 2.

### Measurement of the Cytokine Level in the Plasma of AHE Patients

The level of the following cytokines: IFN-Ɣ, IL-4, IL-10, IL-2, IL-12, and IL-1β was measured in the plasma of AHE patients using ELISA kits (R&D Systems, Minneapolis, MN, United States) according to the manufacturer’s instructions.

### Statistics

Statistical analyses were performed using the GraphPad Prism software 8 (GraphPad Software, La Jolla, United States) using the Mann–Whitney test, unpaired Student’s *t*-test, and one-way ANOVA multiple comparisons. *P* < 0.05 was considered significant. Error bar depicts mean ±SEM, unless others are specified.

## Results

### Assessment of the HEV Load and Positive (+) and Negative (−) RNA Strands in the PBMCs of AHE Patients

**FIGURE 1:**
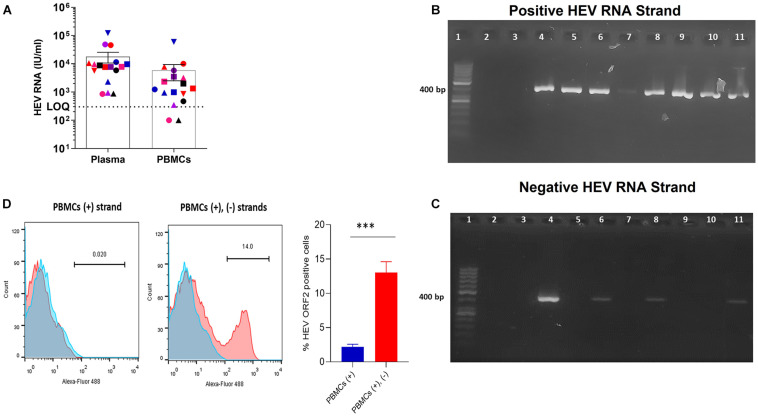
Assessment of the viral load in PBMCs of AHE patients and detection of (+) and (–) RNA strands.**(A)** HEV RNA was quantified in the plasma and PBMCs from AHE patients (*n* = 17). Different colors and symbols represent different patients. The same color and symbol in the different groups (*x*-axis) indicate the same patient. LOQ: limit of quantification. **(B,C)** Representative gel showing the detection of strand-specific (+) HEV RNA strand **(B)** and (–) HEV RNA strand **(C)** in the PBMCs of AHE patients using strand-specific nested PCR targeting HEV ORF1. Lanes 6–11 **(B,C)** show strand-specific RT PCR for the following patients: pt#17 (lane 6), pt#12 (lane 7), pt#11 (lane 8), pt# 10 (lane 9), pt#9 (lane 10), pt#1 (lane 11). Lane 3 in **(B,C)** represents non-template control (negative controls for both assays). Lane (4) in **(B,C)** represents RNA extracted from primary human monocytes infected with HEV-1 *in vitro*, which is a positive control for both (+), and (–) HEV RNA strands using strand-specific primers for each strand. Lane (5) in **(B,C)** represents RNA extracted from the plasma of pt#17, which serves as a positive control for (+) RNA strand and negative control form (–) RNA strand. Lane 1 shows a 50-bp DNA ladder. Lane 2 in **(B,C)**: left empty. **(D)** Representative gating showing the detection of the HEV ORF2 protein in the PBMCs of AHE patients. Blue histograms represent cells treated with the secondary A488 anti-mouse antibodies, and red histograms represent cells stained by the mouse anti-HEV-ORF2 followed by the secondary A488 anti-mouse antibodies. (Left) Expression of HEV ORF2 protein in the PBMCs with detectable (+) HEV RNA strand only (pt # 12). (Middle) Expression of HEV ORF2 protein in the PBMCs with detectable (+), (–) HEV RNA strands (pt # 1). (Right) Percentage of HEV ORF2 positive cells from PBMCs with detectable (+) HEV strand only (*n* = 10) and PBMCs with detectable (+), and (–) HEV RNA strands (*n* = 4). Error bar represents mean ± SEM, ***p < 0.001, as determined by the unpaired *t*-test.

PBMCs were isolated from the blood of AHE patients (*n* = 17) infected with HEV genotype 1. Using RT-qPCR and primers targeting HEV ORF2/3, the median plasma HEV RNA (with IQR) was 7.8 × 10^3^ IU/ml (4.005 × 10^3^–1.0975 × 10^4^ IU/ml), and the median (with IQR) PBMC RNA was 1.34 × 10^3^ IU/ml (6.65 × 10^2^–4.65 × 10^3^ IU/ml) (Figure 1A). HEV RNA was under LOQ in two PBMC samples by RT-qPCR that have a low plasma viral load; 850, 880 IU/ml, respectively (Figure 1A). Then, we assessed the presence of (+) and (−) HEV RNA strands in the PBMCs (*n* = 17) by strand-specific nested RT-PCR targeting HEV ORF1. Positive HEV RNA strands were detected in 14 PBMC samples, from which four samples tested positive for the negative HEV RNA strand (Figures 1B,C and Table 1). The PBMC samples that tested positive for the (−) RNA strand were the ones that showed the highest plasma viral load. PBMCs (*n* = 2, pt#5 and pt#13) tested negative for HEV RNA by RT-qPCR targeting HEV ORF2/3 and also were negative for (+) and (−) RNA strands by nested RT-PCR (Table 1). One PBMC sample (pt#14) was positive (at the LOQ, HEV load: 3.50 × 10^2^ IU/ml) for HEV RNA by RT-qPCR although this sample tested negative by nested RT-PCR for both (+) and (−) HEV RNA strands (Table 1). To assess if the synthesis of the (−) HEV RNA strand in the PBMCs was associated with the production of HEV viral protein, we tested the expression of HEV ORF2 protein in the PBMCs with the detectable (+) strand only (*n* = 10) and the PBMCs with (+), and (−) HEV RNA strands (*n* = 4). Interestingly, we found HEV ORF2 protein is expressed in the PBMCs with (+) and (−) RNA strands, and the mean percentage of HEV ORF2 positive cells was 13% in the four tested patients (Figure 1D) although there was no or very low (less than 3%) expression of HEV ORF2 protein in the PBMCs with the detectable (+) HEV RNA strand only (Figure 1D). No HEV ORF2 protein was recorded in the PBMCs of healthy controls.

**TABLE 1:** Assessment of HEV RNA in the PBMCs of AHE by RT-qPCR and nested PCR.

Patient ID	HEV RNA by RT-qPCR		HEV RNA in PBMCs	
	targeting HEV		by nested RT-PCR	
	ORF2/3 (IU/ml)		targeting ORF1	
	Plasma	PBMCs	(+) strand	(−) strand
Pt#1	+	+	+	+
Pt#2	+	+	+	−
Pt#3	+	+	+	−
Pt#4	+	+	+	+
Pt#5	+	<LOQ^*a*^	−	−
Pt#6	+	+	+	−
Pt#7	+	+	+	−
Pt#8	+	+	+	−
Pt#9	+	+	+	−
Pt#10	+	+	+	−
PT#11	+	+	+	+
Pt#12	+	+	+	−
Pt#13	+	<LOQ^*a*^	−	−
Pt#14	+	+ (at LOQ)	−	−
Pt#15	+	+	+	−
Pt#16	+	+	+	−
Pt#17	+	+	+	+

**^*a*^Means under Limit of quantification (LOQ), LOQ is 300 IU/ml. +, means positive, −: means negative.**

### Evaluation of the Level of Immune Transcripts in the PBMCs of AHE

**FIGURE 2:**
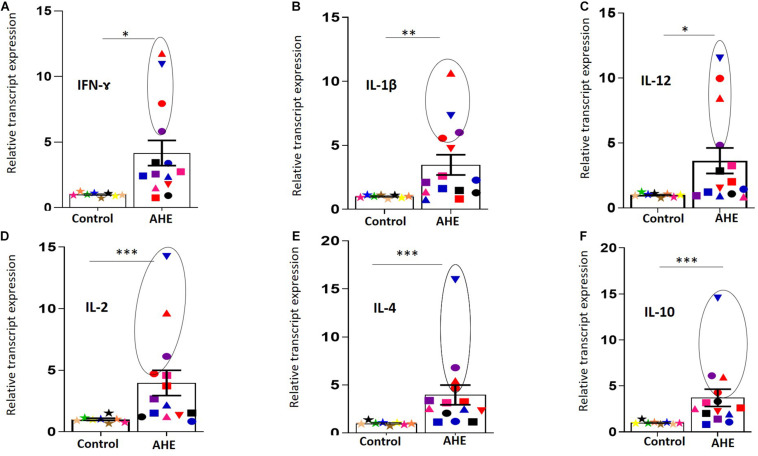
Upregulation of the immune transcripts in the PBMCs of AHE patients.**(A–F)** PBMCs from AHE patients (*n* = 14) and healthy controls (*n* = 8) were assessed for the immune transcripts; IFN-Ɣ **(A)**, IL-1β **(B)**, IL-12 **(C)**, IL-2 **(D)**, IL4 **(E)**, and IL-10 **(F)** by RT-qPCR. The transcript expression for each gene was normalized to the housekeeping gene. Different colors and symbols indicate different patients. Data represented as mean ± SEM. Circle is added around the PBMCs with detectable (+) and (–) RNA strands. *, **, *** indicate that *p* < 0.05, 0.01, and 0.001, respectively, as determined by Mann–Whitney test.

To assess the impact of HEV infection and/or replication in the PBMCs on the induction of immune response, we measured the level of immune transcripts in the PBMCs of AHE. To this end, we included PBMCs from healthy controls (*n* = 8) of comparable age and sex to the AHE patients and compared the level of cytokine transcripts in both healthy controls and AHE patients. We analyzed the transcript levels of cytokines that are involved in both innate and adaptive immune responses, including both humoral and cell-mediated immunity, such as IFN- and IL-1β, IL-12, IL-2, IL-4, and IL-10. We found that the transcript level of these cytokines was significantly upregulated (about four to sixfold) in AHE patients compared with healthy controls (Figures 2A–F).

### Negative HEV RNA Strand in the PBMCs Is Associated With Enhanced Immune Response

**FIGURE 3:**
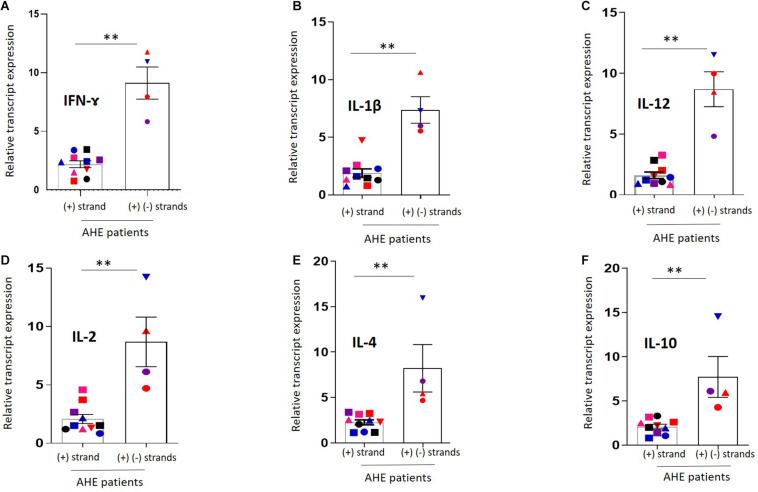
Comparison of the cytokine transcripts between PBMCs with detectable (+) and (–) RNA strands vs. PBMCs with detectable (+) RNA strand.**(A–F)** The immune transcripts including IFN-Ɣ **(A)**, IL-1β **(B)**, IL-12 **(C),** IL-2 **(D)**, IL4 **(E)**, and IL-10 **(F)** were compared between PBMCs with detectable (+) and (–) RNA strands (*n* = 4) versus PBMCs with the detectable (+) RNA strand only (*n* = 10). Data represented as mean ± SEM. ** indicates *p* < 0.01 as determined by Mann–Whitney test.

Then, we compared the level of immune transcripts in AHE patients who tested positive for the (+) HEV RNA strand in the PBMCs (*n* = 10) with AHE patients (*n* = 4) who tested positive for (+) and (−) HEV RNA strands in the PBMCs. Higher cytokine transcripts were recorded in PBMCs of the patients who tested positive for the (−) HEV RNA strand (pt#1, pt#4, pt#11, and pt#17) (Figures 2, 3), and the transcript level of the previous cytokines was significantly upregulated in the AHE patients with detectable (+) and (−) RNA strands in the PBMCs compared with AHE patients with only detectable (+) RNA strand in the PBMCs (Figures 3A–F).

### Assessment of the Cytokines in the Plasma of AHE Patients

**FIGURE 4:**
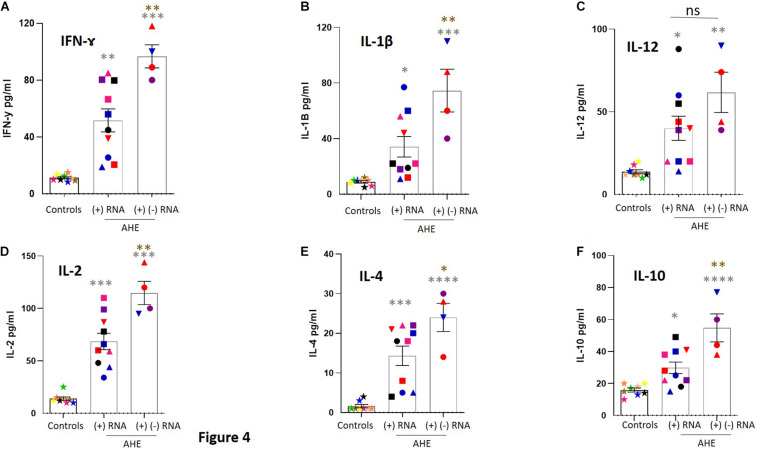
Assessment of the level of cytokines in the plasma of AHE patients. **(A–F)** The level of cytokines, including IFN-Ɣ **(A),** IL-1β **(B)**, IL-12 **(C)**, IL-2 **(D)**, IL4 **(E)**, and IL-10 **(F)**, was measured in the plasma of healthy controls (*n* = 8), plasma of AHE patients who tested positive for the (+) RNA strand in the PBMCs (*n* = 10), and plasma of AHE patients who tested positive for (+) and (–) RNA strands in the PBMCs (*n* = 4). Data represented as mean ± SEM. * (gray); compare AHE vs. healthy control, * (brown): compare AHE patients with detectable (+) and (–) RNA strands in the PBMCs vs. AHE patients with detectable (+) RNA strand in the PBMCs. *, **, ***, and **** means *p* < 0.05, 0.01, 0.001, and 0.0001, respectively, as determined by one-way ANOVA multiple comparison test.

Then, we assessed the level of cytokines in the plasma of AHE patients and compared it with healthy controls. The median plasma IFN-Ɣ, IL-1β, IL-12, IL-2, IL-4, and IL-10 in AHE patients with detectable (+) HEV RNA strands was significantly higher than healthy controls (Figures 4A–F and Table 2). Interestingly, the level of these cytokines, except IL-12, was significantly increased in the plasma of AHE patients with detectable (+), (−) HEV RNA strands in the PBMCs compared with AHE patients with a detectable (+) HEV RNA strand in the PBMCs (Figures 4A–F).

**TABLE 2:** The level of cytokines in the plasma of AHE patients and healthy controls.

Cytokine^*a*^	Healthy controls (*n* = 8)	AHE with detectable (+) HEV RNA strand (*n* = 10)	AHE with detectable (+), (−) HEV RNA strands (*n* = 4)
IFN-Ɣ	10.50 (9.6–13.23)	50.45 (24.28–79.93)	94.50 (82.25–113.5)
IL-1β	10.00 (6.5–10)	22.00 (16.50–57)	74.00 (45–104.5)
IL-12	12.00 (12–17)	39.50 (20–56.25)	59.00 (40.25–86)
IL-2	12.50 (10.50–14.75)	63.00 (47–90)	110.0 (96.25–138)
IL-4	1.000 (1–2.5)	18.00 (5–21.25)	26.00 (16.5–29.5)
IL-10	16.00 (13.25–19.5)	26.50 (21–40.25)	52.00 (39.5–72.75)

**^*a*^All values are expressed as median with IQR, the values of these cytokines expressed as pg/ml.**

### HEV Persistence and/or Replication in the PBMCs and AHE Patients’ Criteria

Then, we asked if HEV persistence [detection of (+) HEV RNA strand] or replication [detection of (+), (−) HEV RNA strands] in the PBMCs is correlated to liver function tests and/or patients’ demographic criteria, such as age and gender. We did not find a significant difference between patients with detectable (+) HEV RNA strands in the PBMCs and patients with detectable (+), (−) HEV RNA strands in the PBMCs in terms of liver function tests, age, and gender (Table 3). However, the liver function tests were slightly elevated in the plasma of patients with detectable (+), (−) HEV RNA strands in the PBMCs than the plasma of patients with a detectable (+) HEV RNA strand in the PBMCs.

**TABLE 3:** Demographic and laboratory characterization of AHE Patients with viral persistence and/or replication in the PBMCs.

	AHE with detectable (+) HEV RNA strand (*n* = 10)	AHE with detectable (+), (−) HEV RNA strands (*n* = 4)	Statistic *P-*value
Age	56 (44–62)	63 (60–70)	0.1661, NS
Gender (M/F)	5/5	2/2	NS
ALT	898 (630–965)	995 (898–1,085)	0.1997, NS
AST	530 (348–640)	700 (605–765)	0.0948, NS
Bilirubin	300 (230–425)	435 (390–518)	0.06, NS

**All values are expressed as median with IQR. NS, non-significant, p > 0.05 as determined by unpaired Student’s t-test*.*

## Discussion

HEV infection causes extrahepatic disorders, including renal disorders, neurological disorders, acute pancreatitis, complications during pregnancy, and hematological manifestations (Pischke et al., 2017; El-Mokhtar and Sayed, 2021). The replication of HEV in hematopoietic stem cells and other blood cells could be a potential source of HEV infection during transplantation (Versluis et al., 2013; Koenecke et al., 2014; Frange et al., 2015; O’Donghaile et al., 2017). In addition, blood products, such as plasma, platelet concentrates, red blood cells, and pooled granulocytes are documented sources of TT HEV infection (Hewitt et al., 2014; Huzly et al., 2014; Gallian et al., 2019). In German blood donation, HEV RNA was detected in the RBCs of all donations in which the viral load in plasma was quantified as > 1,000 IU/m (Dreier et al., 2018). Data about HEV replication in PBMCs is limited. Ippagunta and colleagues report the presence of a (+) HEV RNA strand in 25% (11/44 patients) of the PBMCs of AHE patients, but (–) strand RNA was not recorded in the tested PBMCs (Ippagunta et al., 2011). On the other hand, our group recently reported that stool-derived HEV-1 and HEV-3 are replicating efficiently in human monocytes, macrophages, and bone marrow–derived macrophages isolated from healthy humans *in vitro* (Sayed et al., 2020c). However, in our previous study, we used stool-derived HEV inoculums for infection experiments, which are different than HEV particles circulating in the blood in which the former ones are non-enveloped, and the latter ones are enveloped (Sayed et al., 2017b, 2019). Several studies show that stool-derived HEV inoculums and blood-derived HEV particles are of different properties and infectivity criteria (Sayed and Meuleman, 2017, 2020; Sayed et al., 2017a, 2020b; Ankavay et al., 2019). Therefore, we aimed to assess if HEV particles circulating in the blood persist and/or replicate in the human PBMCs *in vivo* during acute HEV infection and if the replication of HEV in these cells could influence the severity of disease through regulation of the cytokine expression.

In this study, we assessed the viral load in the PBMCs of AHE patients (*n* = 17) infected with HEV-1. We detected HEV RNA in 15/17 (88.23%) and 14/17 (82.3%) of the PBMCs by RT-qPCR targeting HEV ORF2/3 and nested RT-PCR targeting HEV ORF-1, respectively. These findings suggest that HEV can persist inside the PBMCs. Similarly, Ippagunta et al. (2011) report that HEV can persist inside the PBMCs of AHE patients during an outbreak in India. Also, HEV RNA was detected in the PBMCs of miniature pigs inoculated with HEV-3 (Jung et al., 2020). Importantly, we detected a (−) HEV RNA strand, which is an intermediate viral replicative, and HEV ORF2 protein in the PBMCs of 4/17 (23.5%) AHE patients, suggesting that HEV replicates inside the PBMCs during HEV infection. These results are concomitant with our previous findings that show HEV particles are replicating efficiently in the primary human monocytes and macrophages (Sayed et al., 2020c). On the other hand, Ippagunta et al. (2011) report the absence of a (−) HEV RNA strand in the PBMCs of AHE patients using a strand-specific rTth assay. The discrepancy between our finding and Ippagunta et al. (2011) regarding the presence of the (−) HEV RNA strand in the PBMCs could be attributed to the methodology used in the detection of the (−) HEV RNA strand. In this study, we used the methodology first described by Chatterjee et al. (2012) and slightly modified by us (Sayed et al., 2017b), and this was based on using a tag primer for cDNA synthesis, followed by exonuclease I treatment and column purification of the cDNA products. This methodology increases the specificity of the assay and reduces false positive signals without affecting the assay sensitivity (Chatterjee et al., 2012). This assay could detect as few as 10 copies of the (−) HEV RNA strand per reaction; therefore, it is suitable for detecting low levels of HEV replication in cells (Chatterjee et al., 2012). Although the rTth assay used by Ippagunta et al. (2011) shows high specificity and low sensitivity, as reported by the authors, another reason for the discrepancy in the results is the number of samples tested. Ippagunta et al. (2011) assessed the (−) RNA strand in few samples (*n* = 6), and in this study, we assessed the (−) RNA strand in a relatively larger number of samples (*n* = 17), which increases the possibility of detection of the (−) RNA strand. In a parallel line to our finding, we recently detected anti-dsRNA in human monocytes and macrophages infected with stool-derived HEV-1 and HEV-3 *in vitro* (Sayed et al., 2020c). Also, in *in vivo* animal models, such as pigs and rabbits, both (+) and (−) HEV RNA strands were recorded in the lymph node of HEV-infected animals (Williams et al., 2001; Wu et al., 2017; Jung et al., 2020). Because the lymph node contains mainly lymphocytes and macrophages, therefore, these cells are targets for HEV replication. Collectively, our findings suggest that HEV either persists and/or replicates in the PBMCs during the infection.

In this study, we assessed the transcript level of cytokine in the patients’ PBMCs, and we compared the transcript level in AHE patients with detectable both (+), (−) RNA strands with AHE patients with a detectable (+) RNA strand only. As expected, we found the transcript level of IFN-Ɣ, IL-1β, IL-12, IL-2, IL-4, and IL-10 were significantly upregulated in AHE patients compared with healthy controls. In a parallel line, the level of these cytokines was elevated in the plasma of AHE patients compared with healthy controls. Similarly, several reports show an increased level of cytokines, such as IFN-Ɣ IL-1β, IL-12, IL-4, and IL-10 with ongoing AHE infection and HEV-associated acute liver failure (Srivastava et al., 2007; Saravanabalaji et al., 2009; Rathod and Tripathy, 2014; Taherkhani et al., 2015; Wu et al., 2020). Also, our previous study shows that the replication of HEV in human monocytes and macrophages *in vitro* was associated with the induction of innate immune response and increased the release of inflammatory cytokines, such as IFN-Ɣ, IL-1β, IL-12, IL-6, MCP-1, and TNF-α (Sayed et al., 2020c). Interestingly, the degree of upregulation and the plasma level of cytokines were significantly higher in the patients with detectable (+), (−) RNA strands in the PBMCs compared with the patients with the detectable (+) RNA strand in the PBMCs, suggesting the induction of immune response concomitant with HEV replication. However, we did not find a significant difference between AHE patients with detectable (+), (−) RNA strands in PBMCs compared with AHE patients with a detectable (+) RNA strand in PBMCs in terms of age, sex, and liver function tests probably due to the small number of the samples tested. Still, the factors that could lead to viral persistence or replication in the PBMCs are not completely understood. In this study, the (−) RNA strand was recorded in the plasma of patients with higher viremia. Future studies could determine the factors leading to viral replication inside the PBMCs during AHE infection.

Collectively our results confirm and expand previous findings regarding the persistence and replication of HEV in PBMCs. PBMCs can be a reservoir for HEV and a potential source for infection for extrahepatic targets. Also, PBMCs could be a potential source for recurrent HEV infection and the development of chronicity, especially in the settings of immunosuppression. Interestingly, Jung et al. (2020) recently reported that HEV enters various organs via the immune cells. Moreover, the persistence and/or replication of HEV in the PBMCs could spread the infection to other blood components that could be an indirect cause of TT HEV infection. Hewitt et al. (2014) report the risk of TT HEV infection was 100, 50, 40, and 25% for the contaminated granulocytes or fresh plasma, apheresis platelets, pooled platelets, and RBC, respectively. Likewise, other hepatotropic viruses, such as HCV are replicating in the PBMCs, and these cells could be a source for infection to extrahepatic organs (Castillo et al., 2005).

There are some limitations to this study. The number of samples was relatively small. This could explain why we could not find a significant difference between the patients with a detectable (+) HEV RNA strand in the PBMCs and the patients with detectable (+) and (−) HEV RNA strands in the PBMCs in terms of liver transaminases, bilirubin, gender, and age. Future studies including a larger number of samples are needed to assess the impact of HEV replication on the liver function parameters and severity of the disease. Another limitation is the absence of the cell culture system in this study. Due to the limited volume of the samples, we did not have excess infected PBMC pellets to isolate HEV particles and test their infectivity *in vitro*. Future studies could assess the infectivity of the HEV particles isolated from the PBMCs of AHE patients and their roles in the development of chronic infection and/or extrahepatic disorders. Also, due to the sample limitation, we could not characterize the specific cell in the PBMCs where HEV specifically replicates.

## Conclusion

In conclusion, herein, we report for the first time that HEV is replicating in the PBMCs of AHE patients. Positive and/or negative HEV RNA strands and HEV protein were recorded in the PBMCs of AHE patients. HEV replication in PBMCs is associated with the induction of innate and adaptive immune response, which is more pronounced in the PBMCs with the detectable (−) RNA strand.

## Data Availability Statement

All relevant data is contained within the article. The original contributions presented in the study are included in the article/Supplementary Material, further inquiries can be directed to the corresponding author/s.

## Ethics Statement

The studies involving human participants were reviewed and approved by Institutional Review Board (IRB Nos. 17200190 and 17300400) at the Faculty of Medicine, Assiut University, Egypt. The patients/participants provided their written informed consent to participate in this study.

## Author Contributions

IS, ZA, DA, MA-M, MA, MI, AS, and ME-M: design, methodology, and analysis. KK and LA-W: patients source. IS and ME-M: supervision. IS: software, writing first draft. All authors: reviewing and editing the final version of manuscript.

## Conflict of Interest

The authors declare that the research was conducted in the absence of any commercial or financial relationships that could be construed as a potential conflict of interest.
