# Supplementary material for: Hepatitis E Virus Persistence and/or Replication in the Peripheral Blood Mononuclear Cells of Acute HEV-Infected Patients
Source: Front Microbiol. 2021 Jul 16;12:696680. doi: 10.3389/fmicb.2021.696680 (PMC8322848; doi:10.3389/fmicb.2021.696680)
Supplement: Supplementary file 1 [file Data_Sheet_1.docx]

**Supplementary Material and Methods**

**1- Quantification of HEV RNA by RT-qPCR**

HEV RNA was detected and quantified by RT-qPCR using primers targeting a conserved 70bp-region in the HEV ORF2/3 overlap as described before [1; 2; 3; 4]. Briefly, extracted RNA was converted into cDNA using RevertAid First Strand cDNA Synthesis Kit (ThermoFischer Scientific, USA) according to the manufacturer’s instructions. RT-qPCR was performed using Applied Biosystems 7500/7500 Fast (Applied Biosystems, Foster City, CA, USA) and TaqMan™ Universal PCR Master Mix (ThermoFischer Scientific, USA) and the following primers; forward primer (5′-GGTGGTTTCTGGGGTGAC-3′) and reverse primer (5′-AGGGGTTGGTTGGATGAA-3′) and a probe (5′-FAM-TGATTCTCAGCCCTTCGC-TAMRA-3’) as described by Jothikumar et al.[5]. A standard curve was generated and calibrated against the WHO 1^st^ international standard (Paul-Ehrlich Institute, Germany) which has an initial concentration of 250,000 IU/ml was used [6; 7]. Serial dilutions of the reference in a ratio (1/3) were made using PBS and extraction of each dilution (initial volume 200 µl) was done using RNeasy Mini Kit (Qiagen, Germany). According to the cycle threshold (Ct) values of the samples, a standard curve was generated. Linearity was observed within the range of 250,000 to 300 IU/ml. The limit of quantitation (LOQ) in this assay was 300 IU/ml which corresponded to 100% detection. Below the concentration of 300 IU/ml, the linearity of the curve was changed, while the assay could still detect samples with of concentration of 100 IU/ml with a detection rate of 90%. Plasma from AHE patients was quantified according to the standard curve and used in each assay as a positive control and for quantification of unknown samples. For the qPCR of PBMCs, the pellets were precipitated and then dissolved in 200ul of PBS. An equal volume of the plasma and suspended PBMCs in PBS (200ul) were extracted using RNeasy Mini Kit (Qiagen, Germany), and HEV RNA was quantified in the extracted RNA from plasma and PBMCs using the methodology and the standard curve described above.

**2- Detection of positive and negative HEV RNA strands**

HEV RNA was assessed in the PBMCs of AHE patients by strand-specific nested RT-PCR using primers targeting HEV ORF1 for (+), (-) RNA strands. We used the methodology first described by Chatterjee et al [8] and modified by us [3; 9]. We adapted the protocol for the specific detection of (+) and (-) sense HEV RNA strands in the PBMCs enrolled in the study. Briefly, RNA was converted into cDNA using Superscript III enzyme (Life Technologies, USA) and the following primers: 5’- CGGTCATGGTGGCGAATAAYGARAAYGAYTTYTCHGAGTTTG-3’ (for negative strand) and 5’- CGGTCATGGTGGCGAATAARTGTTATTCATTCYAMCCK-3’ (for the positive strand) according to the following conditions: incubation at 55°C for 1 hr, followed by inactivation at 70⁰C for 15 minutes. The cDNA was then treated with Exonulease enzyme (50U), incubate at 37°C for 30 minutes, then inactivation of exonulcease at 85°c for 20 minutes. Then the cDNA was purified using PCR clean up kit (Promega), and then elution in a final volume of 30µl. The first PCR reaction for the (-) HEV RNA strand was done using the following primers: forward: 5’-CGGTCATGGTGGCGAATAA-3’ (tag primer) and reverse: 5’-RTGTTATTCATTCYAMCCK-3’. The first PCR reaction for the (+) HEV RNA strand was done using the following primers: forward: 5’-YGARAAYGAYTTYTCHGAGTTTG-3’ and reverse: 5’-CGGTCATGGTGGCGAATAA-3’. The PCR reaction was done using GoTaq® DNA Polymerase (Promega) according to the following conditions: initial denaturation at 95°C for minutes, followed by 40 cycles of amplifications: denaturation at 95°C for 30 sec, primer annealing at 55°C for 30 sec, extension at 72°C for 1 minute, and final extension at 72°C for 5 minutes. Then, purify the first PCR products using PCR clean up kit (Promega). The second PCR reaction was done using first PCR products, GoTaq® DNA Polymerase (Promega), and the following primers: forward: 5’-AYACYRTYTGGAAYATGGC-3’, and

reverse: 5’-ARCATGCCAATMAGGTTATG-3’ according to the following conditions: initial denaturation at 95°C for minutes, followed by 30 cycles of amplifications: denaturation at 95°C for 30 sec, primer annealing at 55°C for 30 sec, extension at 72°C for 30 sec, and final extension at 72°C for 5 minutes. Then, run the second PCR products on 1% w/v agarose gel. The expected bands should be around 415 bp. Primers used for cDNA, first PCR reaction, and second PCR reaction are mentioned in the supple table (1).

**Supplementary Table 1:** Primers used for specific amplification of positive (+)- and negative (-) HEV RNA strands

|  | (+) Strand RNA | (-) Strand RNA |
| --- | --- | --- |
| cDNA synthesis | 5’- CGGTCATGGTGGCGAATAARTGTTATTCATTCYAMCCK-3’ | 5’- CGGTCATGGTGGCGAATAAYGARAAYGAYTTYTCHGAGTTTG-3’ |
| 1^st^ round PCR | Forward: 5’-YGARAAYGAYTTYTCHGAGTTTG-3’  Reverse: 5’-CGGTCATGGTGGCGAATAA-3’ | Forward: 5’-CGGTCATGGTGGCGAATAA-3’  Reverse: 5’-RTGTTATTCATTCYAMCCK-3’ |
| 2^nd^ round PCR | Forward: 5’-AYACYRTYTGGAAYATGGC-3’  Reverse: 5’-ARCATSCCAATMAGGTTATG-3’ | Forward: 5’-AYACYRTYTGGAAYATGGC-3’  Reverse: 5’-ARCATSCCAATMAGGTTATG-3’ |

Y= C+T, R= A+G, H= A+T+C, M=A+C, K=G+T, S=C+G and 5’CGGTCATGGTGGCGAATAA3’ is a non-HEV tag sequence used as target for the 1st round PCR.

| Gene | Primer sequence 5’=3’ | Product Size (Bp) |
| --- | --- | --- |
| Β-actin | Forward CACCAACTGGGACGACAT  Reverse ACAGCCTGGATAGCAACG | 189 |
| IL- β1 | Forward CCACAGACCTTCCAGGAGAATG  Reverse GTGCAGTTCAGTGATCGTACAGG | 131 |
| IFN-ɤ | Forward GAGTGTGGAGACCATCAAGGAAG  Reverse TGCTTTGCGTTGGACATTCAAGTC | 124 |
| IL-12B subunit | Forward: ACCCTGACCATCCAAGTCAAA  Reverse: TTGGCCTCGCATCTTAGAAAG | 182 |
| IL-4 | Forward: TCTTCCTGCTAGCATGTGCC  Reverse: GACATGCATGCTGCTTGGAG | 162 |
| IL-10 | Forward: GCTGGAGGACTTTAAGGGTTACCT  Reverse: CTTGATGTCTGGGTCTTGGTTCT | 109 |
| IL-2 | Forward: GAATCCCAAACTCACCAGGATGCTC  Reverse: TAGCACTTCCTCCAGAGGTTTGAGT | 115 |

**Supplementary Table 2: Primers used for gene expression in the PBMCs of AHE patients**

**400bp**


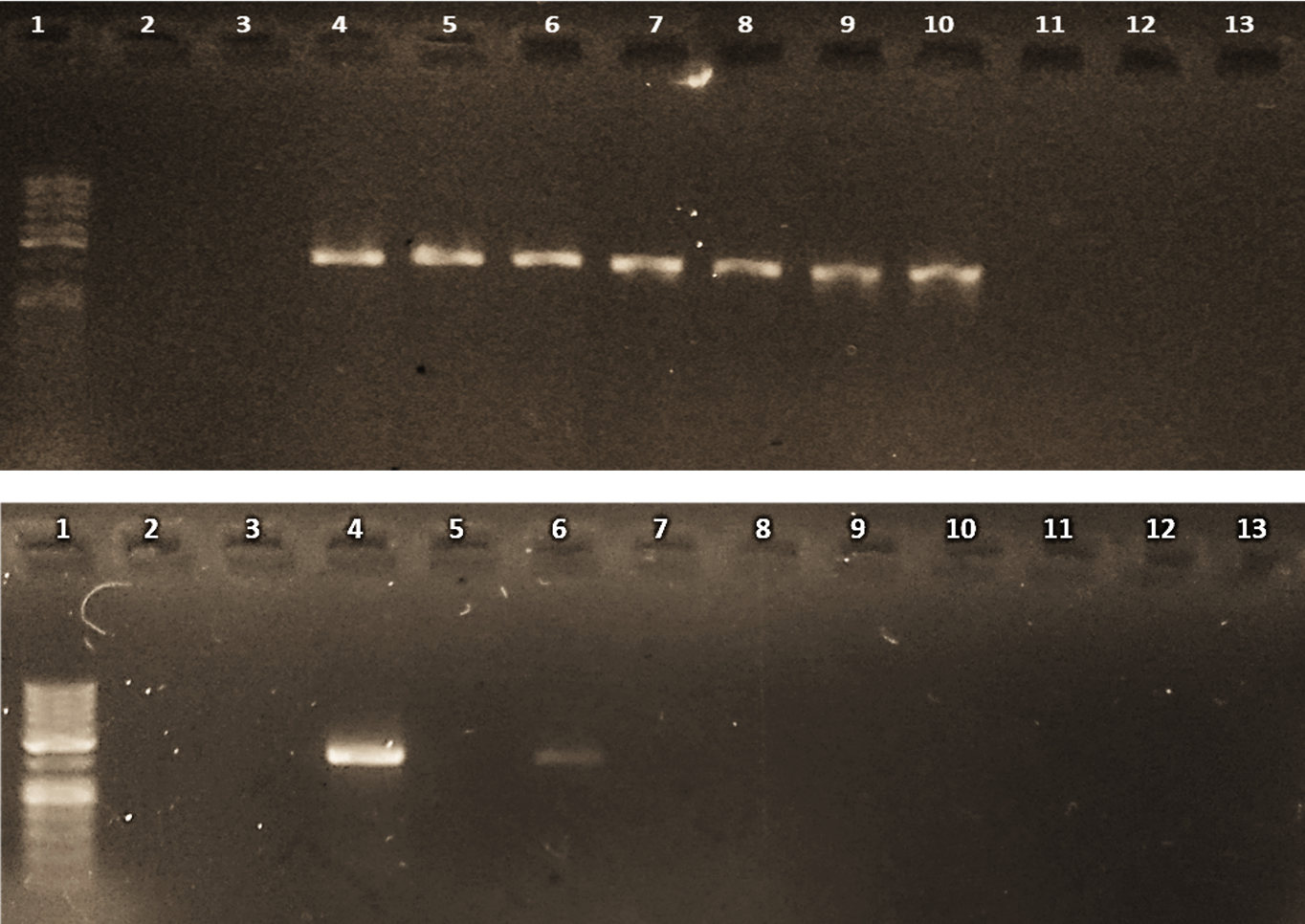


**400bp**

(+) HEV RNA strand

(-) HEV RNA strand

**Figure Legend:** Representative gel showing the detection of strand-specific (+) HEV RNA strand (upper panael) and (-) HEV RNA strand (lower panel) in the PBMCs of AHE patients using strand-specific nested PCR targeting HEV ORF1. Lanes 6-13 (upper, lower panel) show strand specific RT PCR for the following patients: pt#4 (lane 6), pt#2 (lane 7), pt#6 (lane 8), pt#8 (lane 9), pt#7 (lane 10), pt#5 (lane 11), pt#13 (lane 12), and pt#14 (lane 13). Lane 3 in (upper, lower panel): represents non-template control (negative controls for both assays). Lane (4) in (upper, lower panel): represent RNA extracted from primary human monocytes infected with HEV-1 in vitro, which is positive control for both (+), and (-) HEV RNA strands using strand specific primers for each strand. Lane (5) in (upper, lower panel): represents RNA extracted from the plasma of pt#17, which serves as positive control for (+) RNA strand, and negative control form (-) RNA strand. Lane 1 shows a 50 bp DNA ladder. Lane 2 in (upper, lower panel): left empty.

**References**

[1] I.M. Sayed, L. Foquet, L. Verhoye, F. Abravanel, A. Farhoudi, G. Leroux-Roels, J. Izopet, and P. Meuleman, Transmission of hepatitis E virus infection to human-liver chimeric FRG mice using patient plasma. Antiviral research 141 (2017) 150-154.

[2] I.M. Sayed, and P. Meuleman, Murine Tissues of Human Liver Chimeric Mice Are Not Susceptible to Hepatitis E Virus Genotypes 1 and 3. The Journal of infectious diseases 216 (2017) 919-920.

[3] I.M. Sayed, L. Verhoye, L. Cocquerel, F. Abravanel, L. Foquet, C. Montpellier, Y. Debing, A. Farhoudi, C. Wychowski, J. Dubuisson, G. Leroux-Roels, J. Neyts, J. Izopet, T. Michiels, and P. Meuleman, Study of hepatitis E virus infection of genotype 1 and 3 in mice with humanised liver. Gut 66 (2017) 920-929.

[4] I.M. Sayed, A.R.A. Hammam, M.S. Elfaruk, K.A. Alsaleem, M.A. Gaber, A.A. Ezzat, E.H. Salama, A.A. Elkhawaga, and M.A. El-Mokhtar, Enhancement of the Molecular and Serological Assessment of Hepatitis E Virus in Milk Samples. Microorganisms 8 (2020) 1231.

[5] N. Jothikumar, T.L. Cromeans, B.H. Robertson, X.J. Meng, and V.R. Hill, A broadly reactive one-step real-time RT-PCR assay for rapid and sensitive detection of hepatitis E virus. J Virol Methods 131 (2006) 65-71.

[6] S.A. Baylis, K.M. Hanschmann, J. Blümel, and C.M. Nübling, Standardization of hepatitis E virus (HEV) nucleic acid amplification technique-based assays: an initial study to evaluate a panel of HEV strains and investigate laboratory performance. J Clin Microbiol 49 (2011) 1234-9.

[7] S.A. Baylis, J. Blumel, S. Mizusawa, K. Matsubayashi, H. Sakata, Y. Okada, C.M. Nubling, and K.M. Hanschmann, World Health Organization International Standard to harmonize assays for detection of hepatitis E virus RNA. Emerging infectious diseases 19 (2013) 729-35.

[8] S.N. Chatterjee, P.B. Devhare, and K.S. Lole, Detection of negative-sense RNA in packaged hepatitis E virions by use of an improved strand-specific reverse transcription-PCR method. J Clin Microbiol 50 (2012) 1467-1470.

[9] C. Montpellier, C. Wychowski, I.M. Sayed, J.C. Meunier, J.M. Saliou, M. Ankavay, A. Bull, A. Pillez, F. Abravanel, F. Helle, E. Brochot, H. Drobecq, R. Farhat, C.M. Aliouat-Denis, J.G. Haddad, J. Izopet, P. Meuleman, A. Goffard, J. Dubuisson, and L. Cocquerel, Hepatitis E Virus Lifecycle and Identification of 3 Forms of the ORF2 Capsid Protein. Gastroenterology 154 (2018) 211-223.e8.
